# Supplementary material for: The interactome of intact mitochondria by cross-linking mass spectrometry provides evidence for coexisting respiratory supercomplexes
Source: Mol Cell Proteomics. 2017 Dec 8;17(2):216–32. doi: 10.1074/mcp.RA117.000470 (PMC5795388; doi:10.1074/mcp.RA117.000470)
Supplement: Supplemental Data [file supp_RA117.000470_133922_0_supp_23978_fzffwf.zip › spectra_annotation/mito_spectra_annotation/451-1-2-1-12-1.pdf]

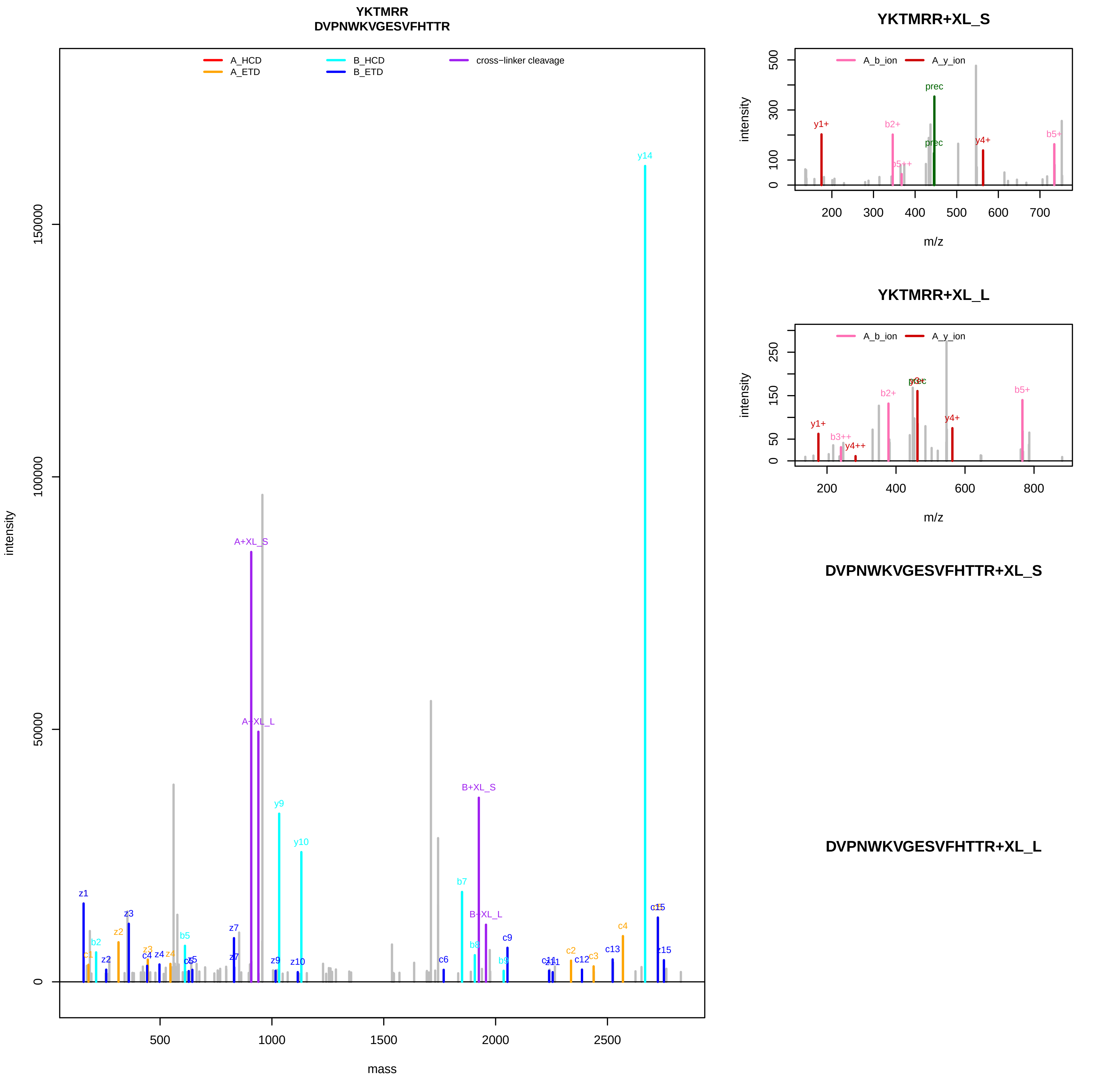
YKTMRR
DVPNWKVGESVFHTTR
A_HCD
A_ETD
B_HCD
B_ETD
cross−linker cleavage
intensity
mass
0
50000
100000
150000
500
1000
1500
2000
2500
y14
A+XL_S
A+XL_L
B+XL_S
B+XL_L
y9
y10
b7
b8
b9
b5
b2
z1
z2
z3
z4
z7
z9
z10
z11
z15
c1
c2
c3
c4
c5
c6
c9
c11
c12
c13
c15
YKTMRR+XL_S
A_b_ion
A_y_ion
prec
y1+
b2+
b5++
y4+
b5+
m/z
0
100
300
500
200
300
400
500
600
700
YKTMRR+XL_L
y1+
b3++
y4++
b2+
y3+
y4+
b5+
0
50
150
250
200
400
600
800
DVPNWKVGESVFHTTR+XL_S
DVPNWKVGESVFHTTR+XL_L
